# Anaplastic Lymphoma Kinase Positive Large B-Cell Lymphoma: Diagnostic Perils and Pitfalls, an Underrecognized Entity

**DOI:** 10.7759/cureus.16882

**Published:** 2021-08-04

**Authors:** Zachariah Chowdhury

**Affiliations:** 1 Pathology, Mahamana Pandit Madan Mohan Malaviya Cancer Centre & Homi Bhabha Cancer Hospital (Tata Memorial Hospital), Varanasi, IND

**Keywords:** anaplastic lymphoma kinase, diagnosis, histopathology, large b cell lymphoma, plasmablastic

## Abstract

Anaplastic lymphoma kinase (ALK) positive large B-cell lymphoma (ALK+ LBCL) is an extremely uncommon non-Hodgkin lymphoma (NHL) with a distinctive histomorphologic, immunophenotypic and cytogenetic profile. It is unlike the more common ALK-positive anaplastic large cell lymphoma, although the latter shares the ALK rearrangement pathognomonic for this entity. ALK+ LBCL is an underrecognized entity since it is rare and unfamiliar, and shares morphologic and immunohistochemical features with a variety of other neoplasms that can result in misdiagnosis. This lymphoma exhibits plasmacytoid morphology and negativity for classical immunomarkers of B- and T-cell lineages, and CD30; however, it expresses terminally differentiated B-cell/plasma cell markers such as CD38, CD138, and MUM-1. Precise identification of this entity is pivotal because of its aggressive behaviour, poor response to standard chemotherapy regimens and the potential for the development of novel targeted therapy. A high index of suspicion on morphology and an extensive immunohistochemistry armoury are required for the veracious detection of this lymphoma, especially at extranodal sites. The purpose of bringing forth this present case, an extranodal neoplasm with plasmacytoid morphology at vertebral location in a young adult, is to highlight the diagnostic perils and pitfalls, the clues to unravel the quandaries and thus, the incredible utility of histopathological examination and immunohistochemical analysis in attaining the unerring diagnosis.

## Introduction

Anaplastic lymphoma kinase positive large B-cell lymphoma (ALK+ LBCL) is an unusual variant of diffuse large B-cell lymphoma (DLBCL) accompanied by exclusive Anaplastic lymphoma kinase (ALK) rearrangements. A tyrosine kinase receptor belonging to the superfamily of insulin receptors, ALK contributes significantly to the development of the brain, and regulates the proliferation of nerve cells [1]. ALK+LBCL displays an immunoblastic or more commonly a plasmablastic histomorphology and an immunoprofile of CD138, ALK, epithelial membrane antigen (EMA), and immunoglobulin (Ig) A expression. Clinically, this lymphoma behaves more aggressively than typical DLBCL with an unfavorable response to conventional chemotherapy [2]. The diagnosis of ALK+LBCL can be taxing due to fewer number of cases having been documented, a dearth of cognizance of this disease, and considerable overlap of morphology and immunoprofile with various haematopoietic and non-haematopoietic tumors. Nonetheless, increased knowledge of the salient features of this neoplasm and thus, appreciation of its existence are crucial for pathologists as well as clinicians, especially keeping in mind the progress in evolving therapeutic remedies. Herein, the author reports a case of this rare disease, and attempts to emphasise the diagnostic pitfalls, and also the pointers to decode the consequential dilemmas.

## Case presentation

**Figure 1:**
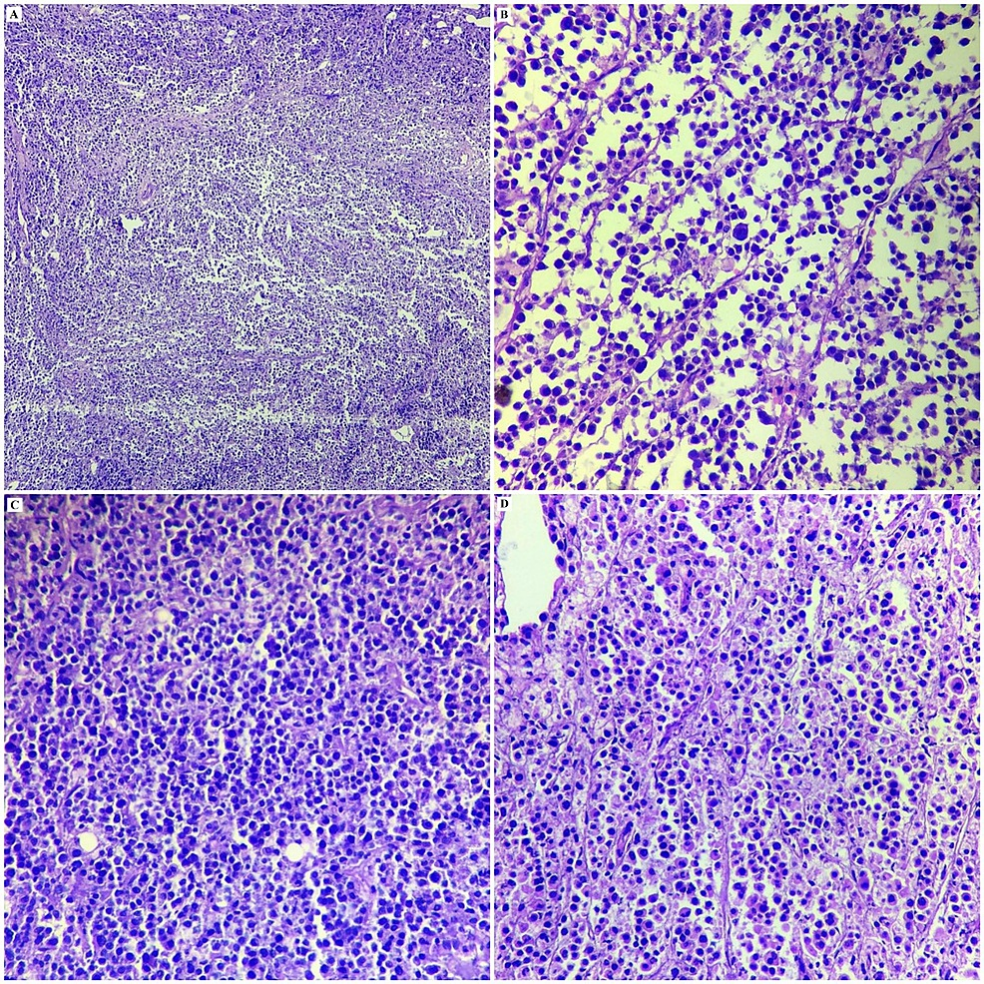
Photomicrograph of the histopathology of ALK+LBCL displaying fibrocollagenous tissue infiltrated by sheets of mostly medium-sized pleomorphic atypical cells separated by fine fibrovascular septa, imparting an alveolar pattern at places. The cells are predominantly plasmacytoid possessing eccentric round to oval nuclei and moderate to abundant cytoplasm, admixed with spotty necrosis [H&E, (A) 10X, (B), (C) & (D) 40X]. H&E: hematoxylin and eosin; ALK+LBCL: anaplastic lymphoma kinase positive large B-cell lymphoma.

An 18-year-old male presented with dull aching abdominal pain for four months and weakness of both lower limbs for three months, accompanied by fever and weight loss. Magnetic resonance imaging (MRI) scan of the thoraco-lumbar spine performed for the complaints of abdominal pain and limb weakness revealed a T2/ST1R hyperintense homogenously enhancing mantle of soft tissues involving the dorsolumbar pre- and para-vertebral regions with epidural, subpleural extension and rib destruction, with the epidural component at D8-D10 levels causing significant cord compression. Bulky bilateral axillary lymphadenopathy measuring 8.6x6.2x12 cm, bulky prevascular lymphadenopathy measuring 2.9x7.7x9.4 cm, along with multiple nodes in the mediastinum and bilateral supraclavicular region were also discovered. Biopsy of the epidural space-occupying lesion at D8-D10 level had been performed and the preliminary histopathology report from a private diagnostic laboratory was small round cell tumor, probably non-Hodgkin lymphoma (NHL). The patient was then referred to our tertiary cancer centre, and the outside reported tissue paraffin blocks and slides were reviewed. Appraisal of the histopathology slides revealed fibrocollagenous tissue infiltrated by sheets of mostly medium-sized atypical cells separated by fine fibrovascular septa imparting an alveolar pattern. The cells were predominantly plasmacytoid possessing eccentric round to oval nuclei and moderate to abundant cytoplasm. Marked pleomorphism was noted at places, with some cells displaying large irregular hyperchromatic nuclei and scant cytoplasm. Focal necrosis was evident (Figure 1).

The differential diagnosis initially considered on histopathological examination (HPE) was broad, encompassing plasmacytoma, small round cell tumors such as rhabdomyosarcoma and Ewing sarcoma, poorly differentiated carcinoma (metastasis), neuroendocrine tumor, melanoma and a host of NHLs, including anaplastic large cell lymphoma (ALCL), DLBCL, plasmablastic lymphoma (PBL) and primary effusion lymphoma (PEL). An elaborate panel of immunohistochemical (IHC) antibodies was executed to attain the diagnosis, which divulged the atypical plasmacytoid cells to be positive for CD138 (diffuse and strong), Mum1 and CD45, and negative for EMA, desmin, CD30, CD20, CD3, CD43, CD56, FLI1, S100, HMB45 and CD79a (Table 1).

**Table 1:** Antibodies used for immunohistochemical analysis.

S. No	Antibody	Dilution	Source
1.	CD138	1:100	Dako
2.	MUM1	1:300	Cell Marque
3.	CD45	1:100	Dako
4.	EMA	1:100	Cell Marque
5.	Desmin	1:100	Dako
6.	CD30	1:100	Cell Marque
7.	CD20	1:200	Dako
8.	CD3	1:100	Dako
9.	CD43	1:100	Cell Marque
10.	CD56	1:50	Cell Marque
11.	FLI1	1:50	Cell Marque
12.	S100	1:500	Cell Marque
13.	HMB45	1:100	Dako
14.	CD79a	1:100	Cell Marque
15.	Kappa	1:50	Cell Marque
16.	Lambda	1:50	Cell Marque
17.	ALK	1:50	Cell marque
18.	CD4	RTU	Cell Marque
19.	Ki67	RTU	Ventana Roche

**Figure 2:**
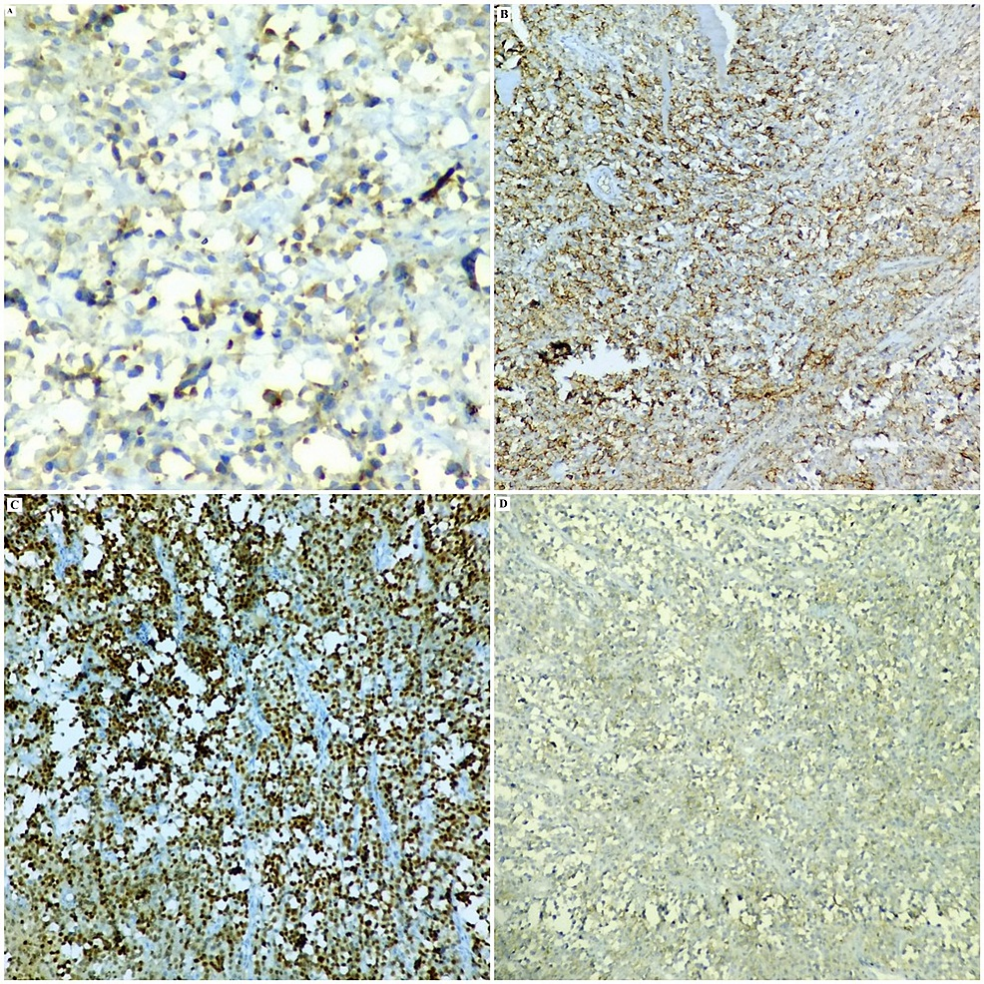
Photomicrograph of the IHC findings of the tumor revealing the tumor cells to be positive for ALK (A, 40X), CD138 (B, 20X), Mum1 (C, 20X) and CD45 (D, 20X). IHC: immunohistochemistry.

**Figure 3:**
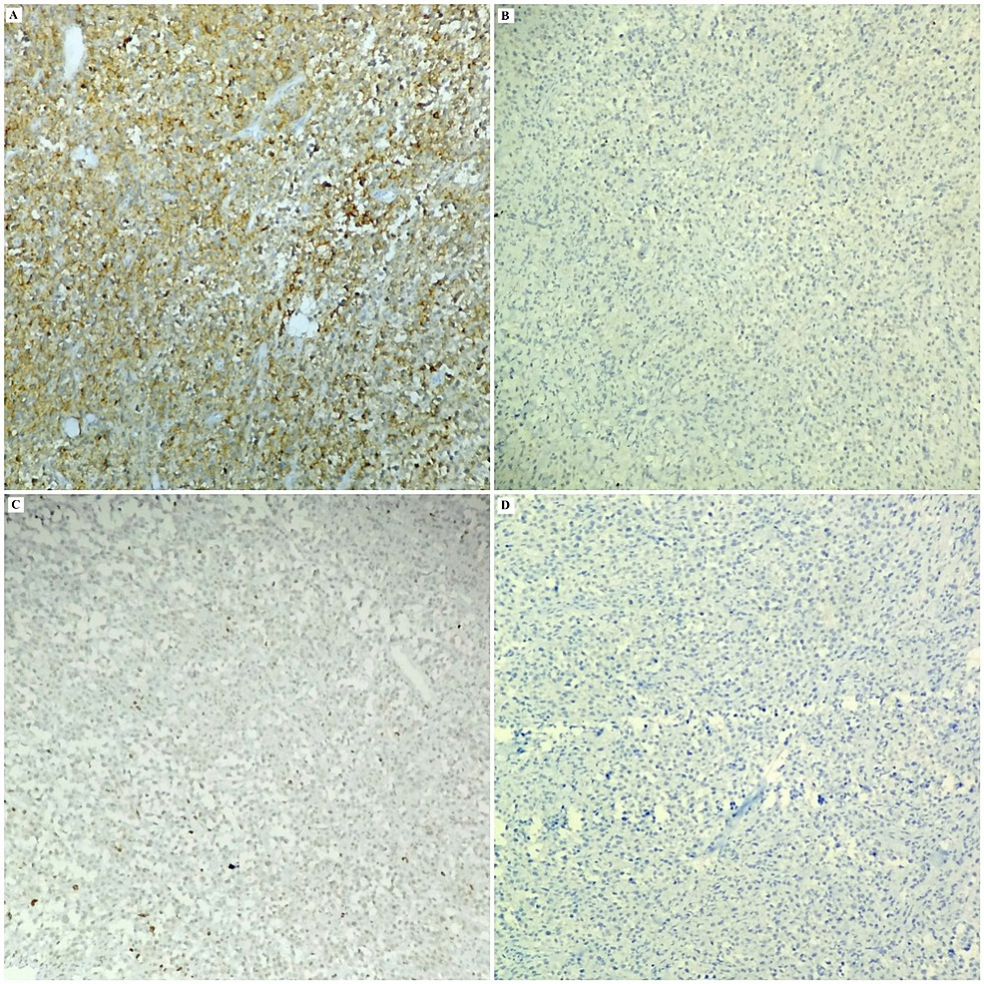
Photomicrograph of the IHC analysis of the tumor displaying the tumor cells to be positive for CD4 (A, 20X) and negative for CD20 (B, 20X), CD3 (C, 20X) and CD30 (D, 20X). IHC: immunohistochemistry.

**Figure 4:**
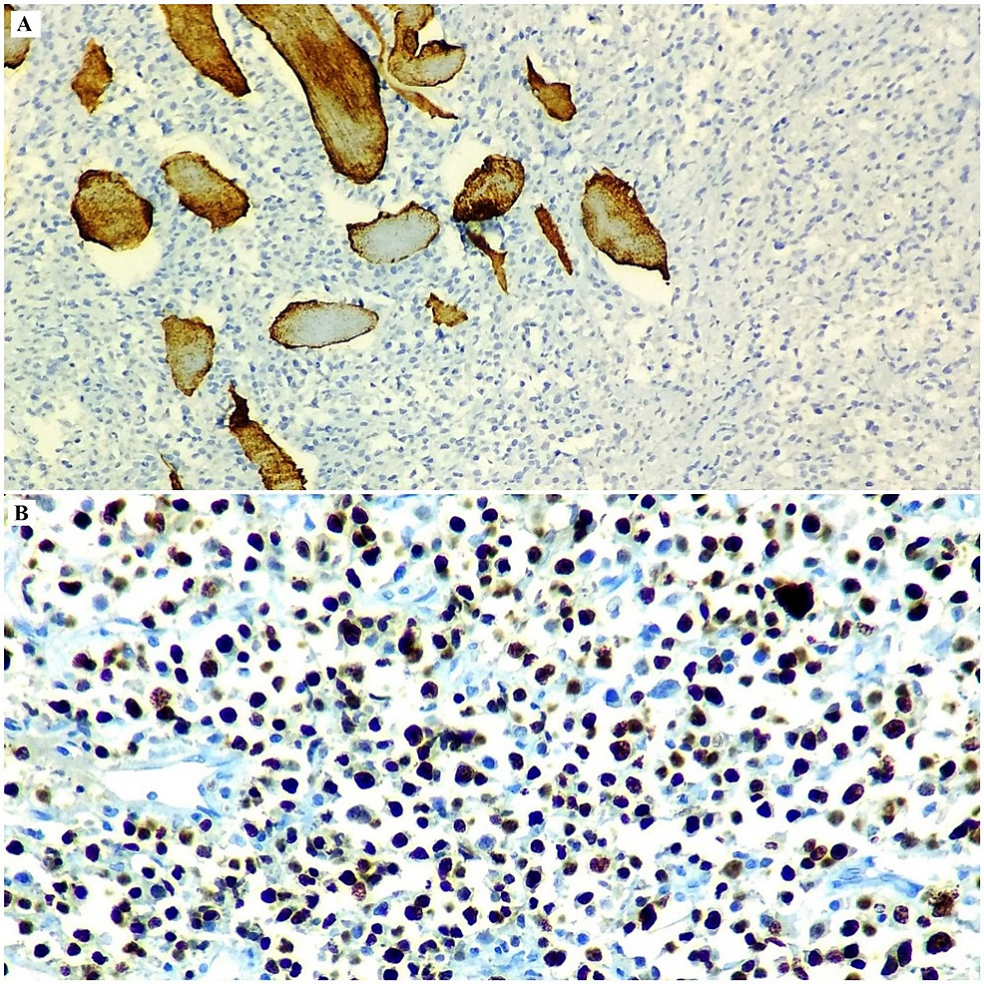
Photomicrograph of the IHC analysis of the tumor exhibiting the tumor cells to be negative for desmin (A, 20X). Ki67 index (B, 40X) was approximately 65-70% in the highest proliferating zone. IHC: immunohistochemistry.

The tumor cells exhibited kappa light chain restriction and Ki67 index of approximately 65%-70%. The diagnosis tilted towards a tumor of plasma cell origin, but the young age of the patient, the significant lymphadenopathy at multiple sites and the immunostaining for CD45 created doubt enough to proceed for a second panel of IHC comprising ALK and CD4. ALK stained the tumor cells diffusely in a cytoplasmic pattern, while CD4 also expressed positivity (Figures 2, 3, 4).

The sample was not subjected to cytogenetic/molecular analysis. Hinging on the histomorphology and the immunoprofile, a final diagnosis of ALK+LBCL was proffered. Bone marrow evaluation did not reveal involvement. Currently, the patient is under treatment protocol, which includes three cycles of CHOP chemotherapy to be followed by radiation therapy.

## Discussion

ALK+ LBCL was originally described by Delsol et al. in 1997 [3]; in an otherwise large series of classical T-/null cell ALK-positive ALCLs, this lymphoma was recognized due to its characteristic lack of CD30 expression. It evinced very aggressive behavior, high relapse rate, and minimal response to standard regimens. This NHL has now been registered as a distinct entity in the 2017 revised edition of the WHO Classification of Haematopoietic and Lymphoid Tissues. Accounting for < 1% of all DLBCLs, it seems to be exceedingly rare and predominantly affects young males, with a male-to-female ratio of 5:1, but does not spare any age group [4]. Primarily a disease of the lymph nodes [3,4,5] or the mediastinum [6-8], extranodal involvement of sites like the tongue [6], nasopharynx [9], stomach [10], liver [4], spleen [4], bone [9], soft tissue [11], and skin [4,12] have been chronicled as well. Generalised lymphadenopathy is the usual presentation, and 60% turn up in advanced stage III/IV. Bone marrow infiltration is observed in approximately one-fourth of the cases [4,12].

Compilation of the histomorphologic features and the immunoprofile, assisted by cytogenetic data when accessible forms the basis of diagnosing ALK+LBCL. In lymph nodes, diffuse or partial effacement of the nodal architecture by the atypical cells can be detected, although initial published reports documented a mainly sinusoidal infiltration. The atypical cells are monomorphic, intermediate to large in size, and flaunt an immunoblastic or plasmablastic morphology with round nuclei, coarse chromatin, single, prominent, central nucleolus and moderate amount of amphophilic cytoplasm. Infrequently, the neoplasm may assume an epithelioid appearance by virtue of a sheeting pattern (more commonly noticed at extranodal sites), and eosinophilic appearance of the cytoplasm. Necrosis, brisk mitosis and a starry-sky appearance may be spotted [4,13,14].

In view of the morphologic overlap with a variety of neoplasms, immunophenotypic analysis is crucial for the diagnosis. The atypical cells are habitually negative for B cell markers - CD20 and CD79a, but parade unflinching positivity for CD138, Mum1 and CD38, compatible with a postgerminal center phenotype, thus simulating a plasma cell neoplasm. However, the most distinctive IHC profile is the positivity for the ALK protein, staining 100% of tumors, with most demonstrating a restricted granular cytoplasmic staining pattern, while the rest display cytoplasmic, nuclear and nucleolar ALK staining [4,15]. CD45, EMA, and MUM-1 are also frequently positive, although CD45 can be weak or even negative [3,4,5,12]. CD4 staining is present in 40-75% of cases [4,13]. IgA with monotypic light chain restriction is consistently observed. Although negative for T cell markers, a few such as CD4, CD43, CD57 and perforin can be positive [4,5,12,13]. CD30 and cytokeratins are negative, although focal and weak staining has been discerned in some cases [3,4,5]. The following markers, though are almost always negative: EBV, CD56, bcl6, bcl2 and human herpes virus 8 (HHV8). Ki-67 proliferation index is documented to range from 50% to 90% [3,4,13-15,16].

The characteristic cytogenetic abnormality is the ALK locus translocation on chromosome 2, and the commonest rearrangement is the t(2;17)(p23;q23), leading to a CLTC-ALK fusion protein. The t(2;5)(p23;q35) translocation is identified in relatively minor cases, resulting in the NPM-ALK fusion [4,9,16]. ALK+ ALCL also demonstrates these two fusion proteins; however, in ALK+ ALCL, NPM-ALK fusion is remarkably more common. Besides, few other genetic events have been described rarely in ALK+ LBCL [4,5,7,12-16]. The presence of the CLTC-ALK fusion protein correlates with granular cytoplasmic ALK staining on IHC as mentioned above, whereas the nuclear, nucleolar and cytoplasmic staining profile is equated with the NPM-ALK fusion product. Thus, the ALK staining pattern can predict the underlying genetic event [4,13,15].

A medley of neoplasms enter the differential diagnoses, hematopoietic and non-hematopoietic as well, especially at extranodal sites. ALK+ LBCL occasionally forms nests and can display round cell morphology and thus, mimic poorly differentiated carcinoma, rhabdomyosarcoma, Ewing sarcoma, neuroendocrine tumors and melanoma; most of these can felicitously be distinguished on the basis of IHC findings, and usually do not cause much headache to the pathologists. However, at times IHC can be fallacious: EMA and occasionally focal cytokeratin are expressed in ALK+ LBCL while the customary B-cell and T-cell markers are not embodied. To boot, CD138 is not specific to the hilt; apart from labelling ALK+ LBCL and plasma cell neoplasm, it also stains the tumor cells in most carcinomas. Hence, in such scenarios, additional IHC with different cytokeratins, other lineage markers, Mum1 and ALK protein ought to resolve the conundrum.

The more arduous quandaries relate to the hematopoietic neoplasms, the most formidable being ALK+ ALCL and plasmacytoma; B cell NHLs disclosing an immunoblastic/plasmablastic morphology, particularly PBL, PEL, HHV8 positive large B cell lymphoma and DLBCL, NOS round off the list. Clinical presentation, sinusoidal template of involvement, and expression of markers like ALK, CD45, EMA (83% in ALK+ ALCL and 93% in ALK+ LBCL), and CD4 (40-70% in ALK+ ALCL and 40-75% in ALK+ LBCL) [4,16,17] are noteworthy overlapping attributes between ALK+ LBCL and ALK+ ALCL. The core distinguishing point is CD30 - diffuse positivity mandated for a diagnosis of ALCL, whereas CD30 is mostly negative in ALK+ LBCL; even in the cases where CD30 exhibits positivity, the staining is focal and weak. ALK+ LBCL is not a T cell neoplasm unlike ALK+ ALCL and hence, lacks rearrangement of clonal T-cell receptor (TCR-gamma or -beta) gene, and instead divulges clonal immunoglobulin (Ig) gene rearrangement. Another notable diagnostic pitfall relates to plasmacytoma including variants such as anaplastic plasmacytoma. There is significant morphologic and immunophenotypic overlap including expression of markers of plasmacytic differentiation. Plasmacytoid appearance of a tumor at extranodal sites, such as vertebra in our case can steer the pathologist towards deducing plasmacytoma, unless one is aware of this rare entity, i.e., ALK+ LBCL and has a high index of suspicion with all the features not conforming to a diagnosis of plasma cell neoplasm. This dilemma can be resolved with the aid of features such as the presence of bone involvement, myeloma component, and lack of ALK and CD4 immunostaining in plasmacytoma. Conversely, the expression of plasma cell markers in ALK+ LBCL accompanied by lack of quintessential B-cell markers and the cytology can mislead to a diagnosis of PBL and/or PEL. PBL and PEL affect HIV-positive patients more often than not and are associated with EBV and HHV8, respectively. On the other hand, immunosuppression, EBV or HHV8 infection have no relation with ALK+ LBCL, and evidence of ALK protein expression excludes PBL, PEL, and HHV8 positive LBCL. Other variants of DLBCL, including DLBCL, NOS can be relatively comfortably segregated from ALK+LBCL due to strong expression of typical B-cell markers such as CD20, CD79a and PAX5 and the truancy of staining for ALK and CD138. Lastly, conspicuous CD4 positivity as observed in the present case should not transpire as a diagnostic peril and lead the pathologist astray towards histiocytic neoplasm and/or myeloid sarcoma. Thus, the plasmablastic morphology/ immunophenotype (CD138, CD38 and Mum1), ALK expression, negativity for CD30 and the non-existence of viral etiologies (EBV, HIV and HHV8) abet the diagnosis of ALK+ LBCL, as illustrated in Table 2.

**Table 2:** Differential diagnosis of ALK positive LBCL: comparative tumor cell immunophenotypes. PDC: poorly differentiated carcinoma; RMS: rhabdomyosarcoma; +: all (or nearly all) cases positive; +/-: majority of cases positive; -/+: minority of cases positive;  -: all (or nearly all) cases negative; ^a^Weak positive; ^b^Can be negative in the small cell variant.

S. No	IHC markers	ALK+ LBCL	ALK+ ALCL	DLBCL, NOS	Plasma-cytoma	PBL	PEL	HHV8-positive DLBCL, NOS	PDC	RMS
1.	LCA	-/+^a^	+/-	+	-/+^a^	-/+^a^	+	+	-	-
2.	EMA	+/-	+/-	-	-	+	+	-	+	-
3.	PanCK	-/+^a^	-/+^a^	-	-	-/+^a^	-/+^a^	-	+	-
4.	CD30	-/+	+/-^b^	+/-	-	+	+	-/+	-	-
5.	ALK	+	+	-	-	-	-	-	-	-
6.	CD138	+	-	-/+	++	++	+	-	+/-	-
7.	MUM1	+	-	+/-	++	++	+	-	-	-
8.	CD3	-	+/-	-	-	-	-/+	-	-	-
9.	CD20	-	-	++	-/+	-/+^a^	-	+/-	-	-
10.	CD79a	-	-	++	-/+	-/+^a^	-	-	-	-
11.	Pax5	-	-/+	++	-/+	-/+^a^	-	-	-	-
12.	Light chain restriction	+	-	+/-	+	+	-/+	+	-	-
13.	CD43	-/+	+/-	-	-	-/+	-/+	-/+	-	-
14.	EBER	-	-	-	-	+	+/-	-	-	-
15.	HHV8 (LANA1)	-	-	-	-	-	+	+	-	-
16.	Desmin	-	-	-	-	-	-	-	-	+

The natural course of ALK+ LBCL is dismal, which is not disparate from other large B-cell lymphomas with plasmablastic differentiation. Promising results have not been reaped with standard lymphoma therapeutics, including CHOP or CHOP-derived chemotherapy with/without radiation and stem cell transplant (autologous as well as allogeneic). This lymphoma being primarily CD20 negative, rituximab is not expected to churn out quantifiable benefits [2,4,8,13,15,16]. Of late, a new class of drugs, namely ALK inhibitors has bagged the spotlight. Patients suffering from relapsed and resistant ALK+ ALCL have proclaimed favorable responses when treated with crizotinib, a small-molecule dual inhibitor of the c-Met and ALK receptor tyrosine kinases. This drug thus, could prove to be a tenable targeted therapy in ALK+LBCL, as well [2,13,15,16,18-20]. Nevertheless, the strongest factor associated with survival in ALK+ LBCL from the literature appears to be the clinical stage at presentation; appreciable longer survival has been observed in patients befalling with localised disease (stage I-II) [4,8,12].

## Conclusions

To conclude, ALK+LBCL is a rare aggressive B-cell NHL with discrete morphologic, immunophenotypic and cytogenetic/molecular findings. In a tumor of plasmacytoid/immunoblastic morphology presenting at nodal/extranodal sites exhibiting immunopositivity for ALK, plasma cell markers and sometimes CD4 and EMA and occasionally focal and weak cytokeratin, and negative staining for both B and T cell markers, CD30 and even CD45 at times, the possibility of this neoplasm should be considered. Owing to the extreme rarity of this entity and consequently lack of a high index of suspicion, morphologic overlap with other hematopoietic and non-hematopoietic neoplasms, unusual immunoprofile and seldom regular employment of ALK IHC, the diagnosis remains challenging and may be missed. Accurate recognition of this entity is of paramount importance, as the promise of a targeted therapy bestows a fascinating recourse for patients with this malady.
